# A comparison of match-physical demands between different tactical systems: 1-4-5-1 vs 1-3-5-2

**DOI:** 10.1371/journal.pone.0214952

**Published:** 2019-04-04

**Authors:** Ivan Baptista, Dag Johansen, Pedro Figueiredo, António Rebelo, Svein Arne Pettersen

**Affiliations:** 1 School of Sport Sciences, University of Tromsø, the Arctic University of Norway, Tromsø, Norway; 2 Computer Science Department, University of Tromsø, the Arctic University of Norway, Tromsø, Norway; 3 Portugal Football School, Portuguese Football Federation, Lisboa, Portugal; 4 Research Center in Sports Sciences, Health Sciences and Human Development, CIDESD, University Institute of Maia, ISMAI, Maia, Portugal; 5 Faculty of Sport, University of Porto, Porto, Portugal; Universita degli Studi di Verona, ITALY

## Abstract

The team tactical system and distribution of the football players on the pitch is considered fundamental in team performance. The present study used time-motion analysis and triaxial-accelerometers to obtain new insights about the impact of different tactical systems (1-4-5-1 and 1-3-5-2) on physical performance, across different playing positions, in a professional football team. Player performance data in fifteen official home matches was collected for analysis. The sample included twenty-two players from five playing positions (centre backs: n = 4; full-back/wide midfielder/ wing-back: n = 9; centre midfielder: n = 6 and centre forward: n = 3), making a total of 108 match observations. A novel finding was that general match physical demands do not differ considerably between these tactical formations, probably because match-to-match variability (variation of players’ running profile from match-to-match) might be higher than the differences in physical performance between tactical systems. However, change of formation had a different impact across playing positions, with centre backs playing in 1-4-5-1 performing significant more HIRcounts than in 1-3-5-2 (p = 0.031). Furthermore, a medium effect size (r = 0.33) was observed in HIRdist, with wide players covering higher distances when playing in 1-3-5-2 than in 1-4-5-1. These findings may help coaches to develop individualised training programs to meet the demands of each playing position according to the tactical system adopted.

## Introduction

To better understand the constraints correlated with sporting success, match analysis has become an important tool in team sports. Nowadays it is well accepted among coaches and sport scientists that the match performance of a football team is, basically, based on four factors: physical, technical, tactical and mental [1]. Even though, the majority of research has been executed within the physical and technical performance domain, previous studies have started to establish connections between physiological demands and tactical behaviour in elite football [2–5].

The lack of research and information about this field can be observed in a systematic review (2012–2016) on match analysis in adult male football [6], where the contextual variables of research analysed (match half, quality of opposition, match location, scoring first, group stage vs knockout phase, substitutions, competitive level and different competitions) did not include the tactical systems used by teams.

The team tactical system and the positioning and distribution of the players on the pitch is considered one of the most important strategic decisions in football [5, 7, 8] and, it is evident that player match-load is influenced by different factors, such as the playing position [2, 9, 10] and the tactical system [11]. This highlights the importance of understanding how physical demands may be affected by playing position in various tactical systems [6]. Despite some previous research [12, 13] addressing the team global positioning on the field, using the measures of centre and dispersion, the role of the tactical system regarding the players’ physical performance, has not been fully described.

Previous studies have concluded that the manipulation of playing formations in small sided games promotes changes in physical performance of teams and players in training [14]. Also, the success of different tactics and strategies depend on the capacities and abilities of the players to perform specific actions during the match. Consequently, players must fulfil the necessary physiological requirements of their playing position inside the tactical system adopted [5, 15, 16].

Previous research has investigated the influence of opposition tactical formation on physiological performance variables and reported higher running distances when playing against a 1-4-2-3-1 formation compared to a 1-4-4-2 formation [17]. In opposition, other studies [11, 18] using various teams and/or different players across different seasons have concluded that tactical systems do not influence the match activity profiles of players. A pilot study with youth players [19] reported no correlation between physical/technical levels and tactical prominence in football matches. However, the identification of the tactical system adopted by a particular team is not a trivial step and previous studies have subjectively defined the tactical formations analysed by using qualified coaches to identify the different formations, as well as to verify if those formations were consistent throughout the game [17, 20]. To the best of our knowledge, no other study has examined the effect of playing formation on player load by position within the same team, in one full season.

An in-depth analysis of match physical performance across playing positions, in different tactical formations, could provide a better understanding of position-specific demands and provide an useful insight to optimize training programs [11]. Therefore, the present study aimed to analyse how tactical systems affect the physical performance of a professional football team across different playing positions in all official home matches during one season. We hypothesize that, despite playing in their specific position, players will accumulate different external workload in matches, depending on the tactical formation deployed.

## Methods

### Participants and match analysis

With institutional ethics approval from UiT The Arctic University of Norway Institutional Review Board, written informed consent from players and approval from the Norwegian Centre for Research Data, data on performance in 15 official home matches from the professional team of a Norwegian elite football club, during one season (2017), was collected for analysis. The matches were all played on artificial grass surface, as described in detail previously [10]. The sample included 22 players (25.2 ± 4.4 years of age; 76.2 ± 6.4 kg of body mass; and, 181.6 ± 5.6 cm of height) across four different playing positions: centre back, CB (n = 4, observations[obs] = 37), full-back/wide midfielder/ wing-back, FB/WM/WB (n = 9, obs = 31), centre midfielder, CM (n = 6, obs = 26), and centre forward, CF (n = 3, obs = 14), making a total of 139 match observations (Table 1). Playing-positions were chosen according to the two tactical formations used by the team and previous research [9, 21, 22]. Team tactical systems and playing positions were determined by two UEFA-qualified coaches (one from the coaching staff of the team analysed) after visualizing video recordings of the sampled matches [17, 20]. These observers subjectively determined the tactical systems used at the beginning of the match and verified if the formations were consistent throughout the matches [17]. Furthermore, 1-4-5-1 and 1-4-3-3 formations were combined, as well as 1-3-5-2 and 1-5-3-2. This procedure was applied due to difficulties in establishing specific differences between similar playing formations when in attacking and defending. When analysing the 1-3-5-2 formation the observers realized that the team often played in 1-5-3-2 formation when not in ball possession (defending) and in 1-3-5-2 with ball possession (attacking). On the other hand, when observing the 1-4-5-1 formation, the observers concluded that the team played in 1-4-5-1 when defending and in 1-4-3-3 when attacking [11, 17]. No other changes in formations throughout the matches were noticed by the observers, therefor no matches were excluded from the analysis.

**Table 1:** Number of match observations per player and tactical system.

Player		*1*	*2*	*3*	*4*	*5*	*6*	*7*	*8*	*9*	*10*	*11*	*12*	*13*	*14*	*15*	*16*	*17*	*18*	*19*	*20*	*21*	*22*
**Observations per tactical system**	**1-4-5-1**	3	5	7	1	3	5	1	0	6	2	4	2	6	0	6	0	1	1	1	0	1	1
	**1-3-5-2**	0	7	7	7	2	0	0	6	5	0	5	3	0	1	6	1	0	0	0	2	0	0
**Total observations**		3	12	14	8	5	5	1	6	11	2	9	5	6	1	12	1	1	1	1	2	1	1

Data was analysed only if: (a) players completed the full match (90 minutes), (b) the player played in the same position during all the match and (c) the team used 1-4-5-1 (1 goalkeeper; 2 CB + 2 FB; 3 CM + 2 WM; 1 CF) or 1-3-5-2 (1 goalkeeper; 3 CB; 3 CM + 2 WB; 2 CF) tactical formations during the entire match.

To ensure players confidentiality, all data was anonymized before analyses.

### Procedures

A stationary radio wave-based Local Positioning Measurement (LPM) tracking system (ZXY Sport Tracking System, Trondheim, Norway), with a default resolution of 20Hz, was used to characterize match activity profiles within the team. Each player wore a specially designed belt, wrapped tightly around the waist, with an electronic sensor system at the player’s lumbar spine, as reported previously [10]. At the stadium, where the matches occurred, there are 6 RadioEyes for optimal coverage, resulting in practically zero packet loss for transponders on the field. If packet loss occurred, the data was linearly interpolated. The accuracy and reliability of the system in measuring player movements in elite soccer competitions have been described in more detail in previous studies [23–25].

### Physical performance variables

Physical parameters analysed included: total distance (TotDist) number of accelerations (acc_counts_), acceleration distance (acc_dist_), number of decelerations (dec_counts_), deceleration distance (dec_dist_), number of HIR (HIR_counts_), HIR distance (HIR_dist_), number of sprints (sprint_counts_), sprint distance (sprint_dist_) and turns.

The HIR (≥19.8 km·h^−1^) and sprinting (≥25.2 km·h^−1^) speed thresholds are similar to those reported in previous research [10, 22, 24, 26].

According to the ZXY Sport Tracking system accelerations were quantified through numerical derivation from positional data with a sampling frequency of 20Hz [25]. Furthermore, accelerations are defined by four event markers: (a) the start of the acceleration event is marked by the acceleration reaching the minimum limit of 1 m·s ^−2^, (b) the acceleration reaches the acceleration limit of 2 m·s ^−2^, (c) the acceleration remains above the 2 m·s ^−2^ for at least 0.5 seconds and (d) the duration of the acceleration ends when it decreases below the minimum acceleration limit (1 m·s ^−2^).

Turns were counted only if the player performed a continuous and significant body rotation of more than 90° in one direction (derived from gyroscope and compass data). The end of a turn and the start of another occurs when a rotation in the opposite direction is measured. The angle threshold used by ZXY Sport Tracking system allowed us to analyse only angles ≥90°.

### Statistical analysis

The results are presented as mean and 95% confidence interval, unless otherwise stated. A linear mixed-effects model with restricted maximum likelihood estimations was used to examine differences in Local Positioning Measurement-derived variables and match duration between 1-3-5-2 and 1-4-5-1 formations. Mixed models can account for unbalanced repeats per player and thus used to model the data. Tactical formation, playing position and their interaction was modelled as fixed effects (effects describing the association between the dependent variable and covariates), while ‘athlete ID’ was included as a random effect (effects generally representing random deviations from the relationships of the fixed part of the model). An α-level of 0.05 was used as level of significance for statistical comparisons. Furthermore, multiple comparisons were adjusted using the Tukey method. The t statistics from the mixed models were converted to effect size correlations [27]. Effect sizes were interpreted as <0.1, trivial; 0.1–0.3, small; 0.3–0.5, moderate; 0.5–0.7, large; 0.7–0.9, very large; 0.9–0.99, almost perfect; 1.0, perfect [28]. All statistical analyses were conducted using the lme4, lsmeans and psychometric packages in R statistical software (version 3.4.1, R Foundation for Statistical Computing, Vienna, Austria).

## Results

### Centre-backs

Slightly higher values, though not statistically significant, were found in HIR_dist_, Acc and Dec (counts and distance), sprint_counts_ and turns when playing in 1-4-5-1 compared to 1-3-5-2 formation (Table 2). Furthermore, CB playing in 1-4-5-1 were observed to perform significant more HIR_counts_ (36.1 ± 3.5) than in 1-3-5-2 (28.2 ± 3.5) (p = 0.008), with a correspondent medium effect size (r = 0.37).

**Table 2:** Mean and 95% confidence interval estimates of different physical parameters from centre backs, analysed according to the tactical system used, and respective p-value and effect size of differences observed (n = 4; observations = 37).

Variables	CB		p-value	Effect Size (r)
	1-4-5-1	1-3-5-2		
TotDist (m)	10865.0 (227.6)	10591.8 (224.0)	0.825	0.15
HIR _counts_	36.1 (3.5)	28.2 (3.5)	0.008	0.37
HIR _dist_ (m)	512.0 (81.5)	431.0 (81.3)	0.658	0.18
Sprint _counts_	6.6 (1.9)	5.4 (1.9)	0.871	0.15
Sprint _dist_ (m)	64.4 (29.6)	74.2 (29.5)	0.999	0.06
Acc _dist_ (m)	325.6 (37.6)	306.9 (37.6)	0.982	0.10
Acc _counts_	63.2 (6.1)	59.7 (6.1)	0.983	0.10
Dec _dist_ (m)	321.2 (41.7)	278.5 (41.6)	0.543	0.20
Dec _counts_	60.3 (6.9)	53.6 (6.9)	0.680	0.18
Turns	32.2 (3.5)	25.8 (3.4)	0.437	0.21

### Wide positions

No significant differences were observed between the tactical formations analysed from players playing in wide positions (Table 3). However, higher values in HIR_dist_ (r = 0.19) and sprint_dist_ (r = 0.16) were found when playing with 1-3-5-2 (977.2 ± 73.7; 236.9 ± 26.8) compared to 1-4-5-1 (838.9 ± 62.5; 195.3 ± 22.7) formation.

**Table 3:** Mean and 95% confidence interval estimates of different physical parameters from full-backs, wide midfielders and wing-backs analysed according to the tactical system used, and respective p-value and effect size of differences observed (n = 9; observations = 31).

Variables	FB/WM/WB		p-value	Effect Size (r)
	1-4-5-1	1-3-5-2		
TotDist	10842.6 (188.8)	11143.0 (233.0)	0.942	0.13
HIR _counts_	45.9 (2.7)	46.9 (3.2)	1.000	0.03
HIR _dist_	838.9 (62.5)	977.2 (73.7)	0.523	0.19
Sprint _counts_	14.1 (1.4)	14.0 (1.6)	1.000	0.01
Sprint _dist_	195.3 (22.7)	236.9 (26.8)	0.747	0.16
Acc _dist_	462.2 (28.5)	447.1 (33.2)	1.000	0.05
Acc _counts_	83.2 (4.7)	76.8 (5.7)	0.950	0.12
Dec _dist_	501.2 (31.5)	505.4 (36.9)	1.000	0.01
Dec _counts_	86.9 (5.3)	86.1 (6.2)	1.000	0.01
Turns	42.1 (2.9)	38.8 (3.7)	0.993	0.11

### Centre midfielders

Small effect sizes were observed in HIR_counts_ (r = 0.12) and Acc_counts_ (r = 0.14) (Table 4), with higher values being observed when playing in 1-4-5-1 (38.5 ± 3.2; 62.3 ± 5.5) than in 1-3-5-2 (35.7 ± 3.4; 55.9 ± 5.9). A similar effect size was also observed in turns (r = 0.15), with CM performing more turns when playing in 1-3-5-2 (40.3 ± 3.7) than in 1-4-5-1 (34.7 ± 3.4).

**Table 4:** Mean and 95% confidence interval estimates of different physical parameters from centre midfielders, analysed according to the tactical system used, and respective p-value and effect size of differences observed (n = 6; observations = 26).

Variables	CM		p-value	Effect Size (r)
	1-4-5-1	1-3-5-2		
TotDist	12009.0 (218.5)	11820.8 (238.7)	1.000	0.09
HIR _counts_	38.5 (3.2)	35.7 (3.4)	0.948	0.12
HIR _dist_	643.2 (73.1)	610.9 (78.1)	1.000	0.06
Sprint _counts_	7.0 (1.6)	7.0 (1.7)	1.000	0.05
Sprint _dist_	101.4 (26.6)	94.8 (28.4)	1.000	0.03
Acc _dist_	313.3 (33.4)	289.6 (35.5)	0.973	0.10
Acc _counts_	62.3 (5.5)	55.9 (5.9)	0.845	0.14
Dec _dist_	358.3 (37.0)	326.0 (39.4)	0.923	0.13
Dec _counts_	69.4 (6.2)	64.2 (6.6)	0.951	0.11
Turns	34.7 (3.4)	40.3 (3.7)	0.782	0.15

### Centre forwards

No significant differences were found regarding any parameter analysed. However, higher values, though with a trivial effect size, in HIR_dist_ and sprint_dist_ can be observed (Table 5) when playing in 1-3-5-2.

**Table 5:** Mean and 95% confidence interval estimates of different physical parameters from centre forwards, analysed according to the tactical system used, and respective p-value and effect size of differences observed (n = 3; observations = 14).

Variables	CF		p-value	Effect Size (r)
	1-4-5-1	1-3-5-2		
TotDist	10724.4 (328.6)	10732.8 (328.6)	1.000	>0.01
HIR _counts_	48.6 (4.7)	47.1 (4.7)	1.000	0.05
HIR _dist_	835.2 (108.5)	930.5 (108.5)	0.881	0.14
Sprint _counts_	11.7 (2.4)	12.8 (2.4)	0.993	0.08
Sprint _dist_	164.5 (39.5)	208.5 (39.5)	0.689	0.18
Acc _dist_	483.4 (49.4)	477.7 (49.4)	1.000	0.02
Acc _counts_	82.9 (8.2)	80.2 (8.2)	1.000	0.05
Dec _dist_	461.4 (54.8)	470.8 (54.8)	1.000	0.03
Dec _counts_	78.3 (9.2)	73.4 (9.2)	0.992	0.09
Turns	36.8 (5.1)	29.7 (5.1)	0.810	0.16

### Tactical system

Significant differences were found in various parameters when comparing the physical performance of the whole team when playing with different tactical systems (Table 6). Significant higher values were observed in HIR_counts_ (r = 0.25) and sprint_counts_ (r = 0.22) when playing in 1-4-5-1 (43.6 ± 1.9; 11.4 ± 1.1) compared with 1-3-5-2 (40.0 ± 2.0; 10.0 ± 1.1) (p = 0.005 and p = 0.015, respectively). Furthermore, when playing in 1-4-5-1, the team was observed to perform more Acc_counts_ (75.8 ± 3.2) and Dec_counts_ (77.8 ± 3.5), as well as covering higher distances in Dec_dist_ (440.3 ± 23.3) than when playing in 1-3-5-2 (71.1 ± 3.4; 72.5 ± 3.6; 413.7 ± 24.2; for Acc_counts_, Dec_counts_ and Dec_dist_) (p = 0.022; p = 0.014 and p = 0.032, respectively).

**Table 6:** Mean and 95% confidence interval estimates of different physical parameters from the whole team, analysed according to the tactical system used, and respective p-value and effect size of differences observed.

Variables	Tactical system		p-value	Effect Size (r)
	1-4-5-1	1-3-5-2		
TotDist	11048.5 (140.2)	11091.2 (149.5)	0.705	0.03
HIR _counts_	43.6 (1.9)	40.0 (2.0)	0.005	0.25
HIR _dist_	779.9 (50.9)	762.8 (52.7)	0.541	0.06
Sprint _counts_	11.4 (1.1)	10.0 (1.1)	0.015	0.22
Sprint _dist_	156.9 (19.1)	158.6 (19.8)	0.867	0.02
Acc _dist_	420.7 (23.1)	401.1 (23.8)	0.085	0.16
Acc _counts_	75.8 (3.2)	71.1 (3.4)	0.022	0.20
Dec _dist_	440.3 (23.3)	413.7 (24.2)	0.032	0.19
Dec _counts_	77.8 (3.5)	72.5 (3.6)	0.014	0.22
Turns	36.9 (1.9)	33.5 (2.0)	0.057	0.16

## Discussion

### Context

The present study provides new insights into the physical demands of two common tactical formations, in elite football players across different playing positions. The context of this study appeared with the change of the head-coach, and consequently, the tactical formation and style of play used of the professional football team analysed. Since this replacement happened in the middle of the season, both tactical formations analysed were composed by an almost equal number of matches (7 and 8 home matches each). It is also important to refer that the change of head-coach led not only to a simple switch of the tactical structure used, but also to a change to a more complex style of play. A more possession and position-oriented style of play were adopted (1-3-5-2) instead of the more direct play and counter-attack strategy used in the first half of the season (1-4-5-1). However, even with all these changes, the context remained the same (same players with similar physical capacities).

### Comparison according to playing position

The results suggest that general match physical demands do not differ considerably between these two tactical formations when compared by playing position. Independent of formation and with few exceptions, players presented similar profiles in all the physical parameters analysed. The most relevant exceptions were the higher HIR_counts_ in CB (1-4-5-1) and longer HIR_dist_ in FB/WM/WB (1-3-5-2), with a medium and small effect size, respectively.

CB playing in 1-4-5-1 performed more HIR_counts_, probably due to the larger area they needed to cover when compared to the area covered by the three CBs when playing in 1-3-5-2. When in defensive organisation (without ball possession), the defensive line of three CBs became most of the time a defensive line composed by 5 players (three CBs and two WBs). The increased number of players playing in the defensive line leads to less m^2^ per player to cover.

Players in wide positions covered more HIR_dist_ when playing in 1-3-5-2 most likely because in this formation the team played with only two wide players (WB), and they needed to cover all the flank, while with 1-4-5-1 formation, those flanks were covered by a total of four players (two on each side).

It has been speculated that match physical demands are higher for CF when playing “alone” in the offensive line (e.g. 1-4-5-1; 1-5-4-1), as they are very often isolated and marked by several opponents [29]. However, the results of the present study are slightly different, since higher, though not significant, values were found in HIR_dist_ and sprint_dist_ for CF, when playing with two attackers (1-3-5-2) compared with playing with only one (1-4-5-1).

Furthermore, no differences in playing time (substitutions) were observed in any playing position between the two tactical systems analysed.

### Comparison according to team workload

When playing position was not taken into consideration and the work-load of the whole team was analysed, the physical workload in some variables was significantly different between tactical systems used. Small significant differences were observed in HIR_counts_ and sprint_counts_, with the team performing more runs (>19,8 km/h) when playing in 1-4-5-1. The number of Acc and Dec was also higher when the 1-4-5-1 system was used. In general, almost all variables analysed presented higher values during the first period of the season (1-4-5-1) than in the second (1-3-5-2).

Previous research [30, 31] has suggested that teams who are winning the match tend to relax and decrease their work-rate. Alternatively, although teams who are losing the match may increase their work-rate during a specified period [32, 33], they may quickly lose the motivation to keep the elevated work rate, which may be especially evident when the goal difference increases negatively (conceding more goals) [34]. In fact, the differences observed between these two tactical systems might be, in part, justified by the significant discrepancy between the score line and match final results achieved during the first and second part of the season. While playing in 1-4-5-1 the team achieved one victory, four draws and three defeats in the eight home matches played. On the other hand, while playing in 1-3-5-2, the team had better results, with five victories, one draw and one defeat in the last seven home matches played. The match results (considerably more draws) and the differences in style of play may therefore, partly justify the higher work-rate of the 1-4-5-1 tactical system.

### Limitations

Our initial hypothesis was that, despite playing in their specific positions, players would accumulate different external workload in matches, depending on the preferred tactical formation. However, the results presented in this study do not fully support the hypothesis, probably because the match-to-match variability might be larger than the differences in physical performance between tactical systems. Like most of the measures in team sports performance, the physical variables used in this study are not stable and are subject to a high variation between successive matches [35]. Furthermore, it has been proved that within-subject (player) and between-match variation in physical performance across the season might be experienced due to changes in the physical condition of the player [36, 37] and environmental conditions [38]. Previous studies have shown that match-to-match variability in performance characteristics of elite soccer players is high [35, 39, 40] and that future research based in match performance requires large sample sizes to identify true systematic changes in workload. In fact, the sample size (22 players/108 observations) might be of such small numbers that true differences can be masked due to a statistical type 2 error, and such a consequence cannot be conclusively ruled out. Previous similar studies have analysed more matches [17] or used considerably larger sample sizes [11] than in the present study. However, they have not compared the physical demands of different tactical systems within the same players in the same context (same team and season) and to do so, a larger sample size than the one used in the present study becomes a difficult task to fulfil.

Even though, the methodology used to determine the team formations is in line with previous studies [11, 14, 17, 20, 41, 42], the process of defining team formations and controlling their consistency throughout the matches was based on the subjective assessment of observers. Further research is needed to attempt to define objectively team formations and to identify when changes occur [17].

Goalkeepers were not included in the present study, however their match activity profiles might be useful and interesting to analyse in different tactical systems and styles of play in future research. All these limitations should be taken into consideration when designing future studies.

### Perspectives and practical application

Since previous research has shown that the players’ physical demands in matches are highly dependent on their positional role in the team [43, 44], analytics, in general, have become a crucial component of team organization and content of training, to meet the position-specific requirements of physical conditioning [45]. This study goes beyond the individualization of training demands according to playing position, also suggesting that the change of tactical system might influence, specific variables of the team’s overall match activity profile, and those differences should be taken into consideration when designing training programs. On the other hand, differences are not notable in all playing positions and these findings should be interpreted with caution, as differences might be team dependent since other teams using the same tactical systems, probably appear with different styles of play.

Change of formation had a different impact on different playing positions, with CB and wide positions presenting more substantial differences than CM and CF. As previously mentioned, the present study and its findings may provide useful and novel insights for coaches on physical performance demands in different tactical formations across playing positions. The information provided should be taken into consideration when designing and implementing training program cycles, according to players’ playing position, the team’s tactical formation and style of play. The individualization and specialization of the training should, therefore, be a matter of reflection and analysis from practitioners.

## Supporting information

S1 File Data review. (XLSX)
